# The Genetic Landscape and Precision Medicine in Neonatal Diabetes Mellitus: From Molecular Mechanisms to Clinical Management

**DOI:** 10.3390/cimb48010104

**Published:** 2026-01-19

**Authors:** Yuanyuan Meng, Lina Zhu, Guanping Dong, Chao Tang

**Affiliations:** Department of Endocrinology, National Clinical Research Center for Children and Adolescents’ Health and Diseases, Children’s Hospital, Zhejiang University School of Medicine, 3333 Binsheng Road, Hangzhou 310052, China

**Keywords:** neonatal diabetes mellitus, monogenic diabetes, precision medicine, K_ATP_ channels, GLIS3, WFS1, sulfonylurea therapy

## Abstract

Neonatal Diabetes Mellitus (NDM) is a rare, heterogeneous monogenic disorder typically presenting within the first six months of life. Unlike type 1 or type 2 diabetes, NDM is caused by single-gene mutations that disrupt pancreatic β-cell function or development. With the advent of next-generation sequencing, the genetic spectrum of NDM has expanded significantly, necessitating a shift from symptomatic management to precision medicine. This narrative review summarizes the genetic basis and pathogenic mechanisms of NDM, categorizing them into three major pathways: (1) ATP-sensitive potassium (K_ATP_) channelopathies (e.g., ABCC8, KCNJ11), where gain-of-function mutations inhibit insulin secretion; (2) Transcription factor defects (e.g., GLIS3, PAX6, GATA6), which impair pancreatic development and often present with syndromic features; and (3) Endoplasmic reticulum (ER) stress-mediated β-cell apoptosis, exemplified by WFS1 mutations. Furthermore, we highlight the clinical complexity of these mutations, including the “biphasic phenotype” observed in ABCC8 and HNF1A variants. Understanding these molecular mechanisms is critical for clinical decision-making. We discuss the transformative impact of genetic diagnosis in treatment, particularly the successful transition from insulin to oral sulfonylureas in patients with K_ATP_ channel mutations, and emphasize the importance of early genetic testing to optimize glycemic control and prevent complications.

## 1. Introduction

Neonatal diabetes mellitus (NDM), often referred to clinically as congenital diabetes mellitus (CDM), represents a rare and complex group of metabolic disorders characterized by persistent hyperglycemia occurring within the first six months of life [1] unlike the more prevalent type 1 diabetes (T1D), which is driven by autoimmune destruction of pancreatic β-cells and involves polygenic susceptibility, NDM is predominantly a monogenic disorder caused by mutations in single genes critical for pancreatic development, β-cell survival, or insulin secretion function [2]. Although the incidence of NDM is low, estimated at approximately 1 in 90,000 to 1 in 160,000 live births, it serves as a unique model for understanding the physiological machinery of human glucose homeostasis [1,3]. Historically, infants presenting with hyperglycemia were treated with lifelong insulin therapy, often under the assumption of early onset autoimmune type 1 diabetes. However, the advent of next-generation sequencing (NGS) technologies over the past two decades has revolutionized our understanding of this condition, revealing a vast landscape of genetic heterogeneity [2]. To date, pathogenic variants in over 30 genes have been identified as causes of NDM. These genes can be broadly categorized into those affecting the ATP-sensitive potassium (K_ATP_) channel function (e.g., KCNJ11, ABCC8), those encoding insulin or involved in its biosynthesis (e.g., INS), and transcription factors that orchestrate pancreatic organogenesis (e.g., GLIS3, GATA6, PAX6, HNF1B) [4,5]. Clinically, NDM is categorized based on the disease course into transient neonatal diabetes mellitus (TNDM), which resolves in infancy but may relapse later in life, and permanent neonatal diabetes mellitus (PNDM), which requires lifelong treatment [6]. However, this clinical dichotomy does not fully capture the molecular complexity of the disease. Recent studies have highlighted that the phenotypic spectrum is heavily dictated by the specific underlying genotype. For instance, mutations in GLIS3 are not only responsible for NDM but also manifest as a syndromic presentation involving congenital hypothyroidism, congenital glaucoma, and renal cysts, reflecting the gene’s pleiotropic role in multiple endocrine organs [7,8]. Similarly, mutations in the WFS1 gene lead to Wolfram syndrome, a severe neurodegenerative disorder characterized by diabetes insipidus, diabetes mellitus, optic atrophy, and deafness (DIDMOAD), caused by chronic endoplasmic reticulum (ER) stress and subsequent β-cell apoptosis [9,10].

The most profound impact of elucidating the genetic basis of NDM lies in the realm of precision medicine. The discovery that activating mutations in the KCNJ11 and ABCC8 genes—which encode the Kir6.2 and SUR1 subunits of the β-cell K_ATP_ channel, respectively—account for approximately 40–50% of PNDM cases has led to a paradigm shift in treatment [2,11]. Patients with these specific channelopathies can often be transitioned from insulin injections to oral sulfonylureas, which bind to the SUR1 subunit to close the K_ATP_ channel and restore endogenous insulin secretion [12]. This “molecular diagnosis-to-treatment” approach has significantly improved glycemic control and quality of life for patients, sparing them the burden of daily injections and reducing the risk of chronic complications [11,13]. Moreover, distinguishing monogenic NDM from type 1 diabetes is crucial for accurate genetic counseling, as recurrence risks vary significantly between de novo mutations, autosomal recessive, and autosomal dominant inheritance patterns [14,15].

Despite these advances, challenges remain. A subset of patients with confirmed NDM still lack a genetic diagnosis, and the interpretation of variants of uncertain significance (VUS) continues to pose clinical dilemmas. Furthermore, the interplay between specific genotypes and long-term extra-pancreatic complications—such as neurodevelopmental delay in DEND syndrome—requires further exploration.

This narrative review aims to provide a comprehensive update on the genetic landscape of congenital diabetes. We analyze the pathogenic mechanisms of key causative genes, including ion channels, transcription factors, and ER stress regulators, and discuss the current strategies for differential diagnosis. Finally, we highlight the implementation of genotype-guided precision therapy, illustrating the paradigm shift from traditional symptom-based management to mechanism-based care.

## 2. Pathogenic Mechanisms and Genetic Classification

### 2.1. Ion Channel Defects (The Channelopathies)

**Figure 1:**
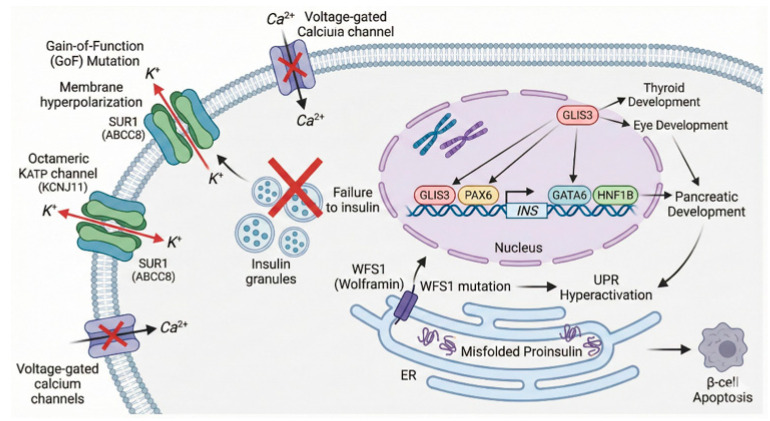
Molecular Landscape of β-cell Failure in Neonatal Diabetes.

The precise regulation of insulin secretion is dependent on the electrophysiological state of the pancreatic β-cell membrane, a process governed centrally by the ATP-sensitive potassium (K_ATP_) channel. This channel acts as a metabolic sensor, coupling cellular energy status to membrane potential. Structurally, the K_ATP_ channel is a hetero-octameric complex composed of four pore-forming subunits (Kir6.2) encoded by KCNJ11, and four regulatory sulfonylurea receptor 1 subunits (SUR1) encoded by ABCC8 [16]. Under physiological conditions, glucose metabolism elevates the intracellular ATP/ADP ratio, inducing channel closure. This blockade prevents potassium efflux, leading to membrane depolarization, the opening of voltage-gated calcium channels, and subsequent calcium-dependent exocytosis of insulin granules [17]. Consequently, genetic disruptions in the subunits of this channel constitute the most common etiology of permanent neonatal diabetes mellitus (PNDM) [16], as shown in Figure 1. This schematic illustrates the three primary pathogenic mechanisms underlying NDM. (Left) Ion Channel Defects: The KATP channel (composed of Kir6.2 and SUR1 subunits) regulates insulin secretion. Gain-of-function (GoF) mutations prevent channel closure, leading to membrane hyperpolarization and inhibition of insulin release. This defect can be bypassed by sulfonylureas, which bind SUR1 to force closure. (Right) Transcription Factor Defects: Mutations in nuclear transcription factors (e.g., GLIS3, PAX6, GATA6, HNF1B) disrupt the transcriptional hierarchy required for pancreatic organogenesis and β-cell differentiation, often leading to pancreatic hypoplasia or agenesis. (Bottom) ER Stress: Mutations in WFS1 (encoding Wolframin) disrupt endoplasmic reticulum (ER) homeostasis and calcium signaling, causing the accumulation of misfolded proinsulin (ER stress), mitochondrial dysfunction, and progressive β-cell apoptosis.

#### 2.1.1. Gain-of-Function Mutations and Insulin Deficiency

The prevailing mechanism underlying KATP channel-related congenital diabetes is the “gain-of-function” (GoF) mutation. Pathogenic variants in either KCNJ11 or ABCC8 reduce the channel’s sensitivity to ATP-mediated inhibition or increase its intrinsic opening probability [16]. As a result, the channel remains constitutively open even in the presence of hyperglycemia and elevated intracellular ATP. This “locked-open” state results in persistent membrane hyperpolarization, thereby abolishing glucose-stimulated insulin secretion (GSIS) despite preserved insulin synthesis [17].

Clinical genotype–phenotype correlations in these channelopathies are robust but variable. Mutations in KCNJ11 are frequently associated with PNDM and, in severe cases, the DEND syndrome (Developmental delay, Epilepsy, and Neonatal Diabetes). This syndromic presentation arises because Kir6.2 subunits are also expressed in neuronal tissues and skeletal muscle, where the permanently open channels affect neuronal excitability and motor function [18]. Conversely, ABCC8 mutations are more commonly linked to transient neonatal diabetes mellitus (TNDM), although they also account for a significant proportion of permanent cases [19].

#### 2.1.2. The Spectrum from Loss-of-Function to Paradoxical Diabetes

While GoF mutations cause diabetes via hyperpolarization, loss-of-function (LoF) mutations in ABCC8 or KCNJ11 traditionally result in the opposite phenotype: congenital hyperinsulinism (CHI). In CHI, the channel fails to open or is absent from the membrane, leading to constitutive depolarization and excessive insulin release [16,20].

However, the dichotomy between “GoF causing diabetes” and “LoF causing hyperinsulinism” has been challenged by recent findings. Emerging evidence describes a “paradoxical” phenomenon where specific LoF mutations, initially causing hyperinsulinemic hypoglycemia in infancy, predispose individuals to glucose intolerance or frank diabetes in later life, often classified as Maturity-Onset Diabetes of the Young (MODY) subtype 12 (associated with ABCC8) [21,22]. Therapeutic strategies for these channelopathies are summarized in Table 1.

Two primary mechanisms have been proposed for this transition. First, persistent membrane depolarization and calcium influx may lead to β-cell exhaustion and apoptosis over time (“burnout” effect) [23]. Second, specific mutations, such as the Ser118Leu variant in KCNJ11, have been shown to reduce channel membrane expression. While this initially mimics a LoF state (hyperinsulinism), the long-term failure of β-cells to rest or respond dynamically to metabolic demand results in progressive functional decline, manifesting as sulfonylurea-sensitive diabetes in adulthood [17]. Similarly, in cohorts of ABCC8-MODY patients, inactivating variants have been identified, confirming that reduced channel activity can paradoxically lead to insulin-deficient phenotypes later in life [22,24].

#### 2.1.3. Therapeutic Implications of Channelopathies

The molecular characterization of KCNJ11 and ABCC8 mutations has arguably provided the most significant success story in precision medicine for diabetes. Since the defect lies in the failure of the channel to close in response to ATP, sulfonylureas—which bind to the SUR1 subunit with high affinity—can bypass the metabolic defect and force channel closure [25]. Large cohort studies have demonstrated that approximately 90% of patients with KCNJ11- or ABCC8-related PNDM can successfully transition from insulin therapy to oral sulfonylureas [16]. Notably, for patients with MODY12 caused by ABCC8 variants, individualized treatment plans incorporating sulfonylureas have shown significant efficacy, allowing some patients to discontinue insulin therapy completely [24].

### 2.2. Transcription Factor Defects: The Architects of Pancreatic Development

While ion channel defects disrupt the real-time function of insulin secretion, mutations in transcription factors cause diabetes by impairing the embryological development of the pancreas and β-cell differentiation. Because these factors—including GLIS3, PAX6, GATA6, HNF1B, and NEUROG3—are pleiotropic regulators active in multiple organ systems, their deficiency rarely causes isolated diabetes. Instead, they present as distinct multi-system syndromes. Recognizing the specific pattern of extra-pancreatic malformations is the key to identifying the underlying genetic etiology.

#### 2.2.1. GLIS3 and the Pancreas-Thyroid-Eye Axis

GLIS3 acts as a master regulator of β-cell generation and thyroid hormone biosynthesis. Biallelic loss-of-function mutations result in a hallmark triad known as NDH Syndrome: Neonatal Diabetes, Congenital Hypothyroidism, and Congenital Glaucoma [26].

Crucially, the clinical spectrum has recently expanded [27,28]. Beyond the classic triad [29], GLIS3 variants are linked to hepatic complications (cholestasis, hepatomegaly) and renal cystic dysplasia, reflecting the gene’s role in maintaining ductal architecture in the liver and kidney [30]. Ophthalmological features can also include high hyperopia, a specific marker distinguishing this from other hereditary glaucomas. Management requires a multidisciplinary approach addressing lifelong insulin replacement, thyroxine therapy, and monitoring for progressive renal and ocular damage [27,31].

#### 2.2.2. PAX6: Aniridia and Defective Insulin Processing

PAX6 is essential for ocular development and islet cell identity. Unlike the structural agenesis seen in other factors, PAX6 mutations cause diabetes via a dual mechanism [32]: reduced β-cell mass and a specific defect in proinsulin processing Patients classically present with aniridia (absence of the iris), which serves as an immediate diagnostic clue [33].

Metabolically, these patients exhibit disproportionately elevated proinsulin levels, indicating a failure in the conversion to mature insulin. This “processing defect” leads to glucose intolerance that may precede frank diabetes. Clinicians should view PAX6 deficiency as a complex metabolic syndrome that may also encompass obesity and thyroid dysfunction, requiring broad systemic surveillance [34].

#### 2.2.3. GATA6 and Pancreatic Agenesis

GATA6 haploinsufficiency is the leading cause of pancreatic agenesis or severe hypoplasia. Because GATA6 is critical for cardiac outflow tract formation, the clinical presentation is dominated by a triad of Neonatal Diabetes, Pancreatic Exocrine Insufficiency, and Congenital Heart Defects (most commonly Tetralogy of Fallot or atrial septal defects). The diabetes is insulin-dependent from birth due to critical β-cell mass reduction. However, unlike autoimmune type 1 diabetes, these patients require pancreatic enzyme replacement therapy to manage malabsorption, a distinction that underlines the importance of accurate genetic diagnosis [13].

#### 2.2.4. HNF1B: The Renal-Cysts and Diabetes Syndrome (RCAD)

HNF1B mutations (MODY5) exemplify the variable expressivity of transcription factor defects [35]. The hallmark is Renal Cysts and Diabetes (RCAD) syndrome. While diabetes may present in the neonatal period, structural renal anomalies (cystic dysplasia, single kidney) often appear prenatally and are the primary diagnostic alert [36]. Recent genotype–phenotype studies have highlighted that genital tract malformations (e.g., uterine anomalies) and electrolyte disturbances, specifically hypomagnesemia, are frequent comorbidities. Therefore, the discovery of renal cysts in a diabetic infant mandates HNF1B testing and screening for associated magnesium deficiency [37].

Although often classified under MODY (MODY5), HNF1B mutations frequently present in the neonatal period or early childhood. HNF1B is vital for the development of the kidney tubules and pancreatic bud. Consequently, the hallmark of HNF1B-related disease is the presence of renal cysts, often detectable prenatally, alongside diabetes, which involves both insulin deficiency and pancreatic atrophy [38]. Genotype–phenotype studies have shown significant variability, with some patients presenting primarily with renal failure and others with isolated diabetes or genital tract malformations [39].

In summary, defects in these transcription factors cause diabetes through the fundamental failure of pancreatic development or cellular differentiation. Unlike channelopathies, these forms of diabetes are typically insulin-dependent and do not respond to sulfonylureas. However, recognizing the specific genetic etiology is critical for predicting and managing the associated multi-system comorbidities, such as monitoring for glaucoma in GLIS3 patients or cardiac defects in GATA6 carriers.

#### 2.2.5. NEUROG3 and Enteroendocrine Dysgenesis

NEUROG3 (Neurogenin 3) acts as the pivotal switch for endocrine differentiation in the gut and pancreas. Biallelic mutations in NEUROG3 cause a rare syndrome characterized by NDM and congenital malabsorptive diarrhea [40]. The diarrhea results from a complete lack of intestinal enteroendocrine cells, leading to the malabsorption of nutrients, which can be life-threatening if not managed with parenteral nutrition. Recent genomic analyses using CUT&RUN technology have mapped extensive NEUROG3 occupancy in the human pancreatic genome, revealing its role in regulating a vast network of genes essential for islet cell identity [41]. Novel variants in NEUROG3 continue to expand the phenotypic spectrum, with some patients presenting with milder, late-onset gastrointestinal symptoms, underscoring the importance of considering this gene in diabetic patients with unexplained chronic diarrhea [42].

### 2.3. Endoplasmic Reticulum Stress: The WFS1 Paradigm

Pancreatic β-cells are uniquely susceptible to endoplasmic reticulum (ER) stress due to their immense insulin biosynthetic burden [20]. This vulnerability is the central pathogenic mechanism in Wolfram syndrome, caused by mutations in the WFS1 gene encoding Wolframin.

Wolframin regulates the Unfolded Protein Response (UPR) and maintains ER-mitochondria calcium transfer. Its deficiency triggers a catastrophic cascade: chronic UPR hyperactivation, mitochondrial dysfunction, and eventual β-cell apoptosis. Clinically, this manifests as DIDMOAD (Diabetes Insipidus, Diabetes Mellitus, Optic Atrophy, and Deafness).

Crucially, because this mechanism involves progressive cellular failure rather than developmental absence, it offers a therapeutic window. Emerging evidence suggests that GLP-1 receptor agonists can mitigate ER stress and preserve remaining β-cell function, shifting the management paradigm from pure insulin replacement to disease-modifying therapy [43].

#### 2.3.1. WFS1 and the Wolframin Protein

The WFS1 gene encodes wolframin, a transmembrane glycoprotein localized primarily to the ER membrane. Wolframin is a crucial regulator of the ER stress response and cellular calcium homeostasis [44]. It functions by negatively regulating the ATF6 branch of the UPR and interacting with the sarcoplasmic/endoplasmic reticulum-ATPase (SERCA) pump to maintain high levels, which are essential for proper protein folding [45].

#### 2.3.2. Pathogenesis: The ER–Mitochondria Axis Failure

In patients with WFS1 mutations, the loss of functional wolframin leads to a catastrophic cascade of cellular failures. First, the absence of wolframin results in chronic, unresolved ER stress, characterized by the hyperactivation of the UPR and the accumulation of misfolded proinsulin [46]. Second, the disruption of homeostasis impairs mitochondrial function. Wolframin is enriched at mitochondria-associated ER membranes (MAMs), contact sites critical for transfer from the ER to mitochondria [47]. WFS1 deficiency disrupts this crosstalk, leading to mitochondrial dysfunction, energy depletion, and the generation of reactive oxygen species (ROS) [48]. The convergence of ER stress and mitochondrial failure ultimately triggers apoptotic pathways in β-cells, causing progressive insulinopenia and diabetes [49].

#### 2.3.3. Clinical Spectrum: Wolfram Syndrome and Beyond

The classic phenotype associated with WFS1 mutations is Wolfram syndrome (DIDMOAD), a rare autosomal recessive neurodegenerative disorder characterized by Diabetes Insipidus, Diabetes Mellitus, Optic Atrophy, and Deafness [43]. Diabetes mellitus is typically the first manifestation, often presenting in the first decade of life, followed by optic atrophy and sensorineural hearing loss. The neurodegeneration observed in these patients mirrors the β-cell destruction, as neurons are also highly dependent on proper ER and mitochondrial function [50].

Recent genotype–phenotype analyses have revealed significant heterogeneity. While nonsense and frameshift mutations typically result in severe, full-blown Wolfram syndrome, missense mutations can present with milder or incomplete phenotypes. For instance, specific WFS1 missense variants have been linked to isolated autosomal dominant diabetes or non-syndromic hearing loss, without the devastating neurodegeneration seen in classic cases [51]. Furthermore, novel splicing variants affecting the acceptor splice site have been identified, which uncover the impact of alternative splicing on β-cell apoptosis, expanding the known pathogenic mechanisms [49].

#### 2.3.4. Therapeutic Horizons: Targeting ER Stress

Unlike channelopathies, diabetes caused by β-cell destruction has traditionally been considered irreversible, requiring lifelong insulin therapy. However, the elucidation of the ER stress mechanism has opened new therapeutic avenues. Preclinical studies using induced pluripotent stem cell (iPSC)-derived β-cells from Wolfram syndrome patients have shown that GLP-1 receptor agonists (e.g., liraglutide) can mitigate ER stress and prevent apoptosis [52]. These agents appear to stabilize cellular function by enhancing mitochondrial bioenergetics and reducing the UPR burden. A recent study demonstrated that liraglutide treatment reversed unconventional cellular defects in WFS1-mutant β-cells, suggesting that repurposing existing diabetes drugs could offer a disease-modifying strategy for this orphan disease [52,53].

In conclusion, WFS1-related diabetes represents a prototype of “conformational diseases” where protein misfolding drives organ failure. Recognizing this mechanism is vital, as it shifts the focus from simple glucose replacement to therapies that support β-cell survival and protect neuro-sensory tissues.

## 3. Clinical Significance: The Syndromic Map and Biphasic Phenotypes

### 3.1. Distinguishing Isolated from Syndromic Diabetes

**Figure 2:**
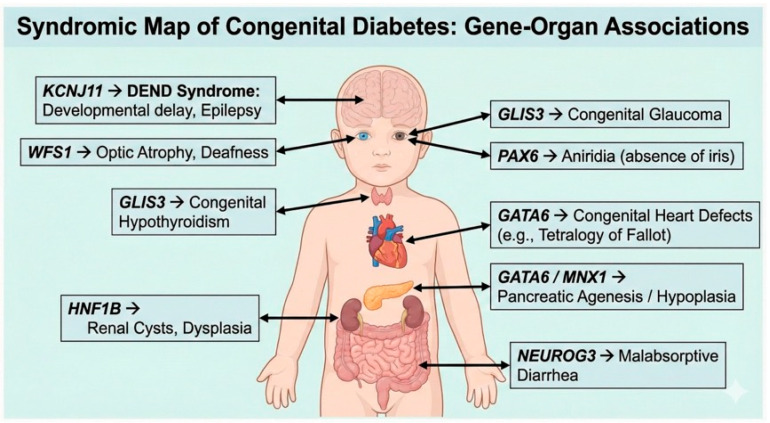
Syndromic Map of Congenital Diabetes: Gene–Organ Associations.

The clinical presentation of congenital diabetes ranges from isolated hyperglycemia to complex multi-system disorders. Distinguishing between these forms is the cornerstone of the diagnostic algorithm (see Table 2 and Figure 2) [54]. Isolated Diabetes: Primarily caused by defects in the functional machinery of the β-cell (e.g., KCNJ11, ABCC8, INS). These patients typically present with DKA or hyperglycemia but lack structural organ defects. While functional neurological features (e.g., DEND syndrome) can occur with channelopathies, the anatomy of the pancreas and other organs is usually normal. Syndromic Diabetes: Arises from mutations in pleiotropic transcription factors (GLIS3, GATA6, HNF1B) or metabolic transporters (SLC19A2, WFS1). As detailed in Section 2, the diabetes is merely one component of a broader developmental failure. Diagnostic Signposting: The presence of specific extra-pancreatic features serves as a powerful “diagnostic filter” to narrow the candidate genes before sequencing results are available: Megaloblastic Anemia + Deafness: Strongly suggests Thiamine-Responsive Megaloblastic Anemia (TRMA) caused by SLC19A2 mutations. This is a critical diagnosis to catch [55], as high-dose thiamine is an effective treatment. Distinct Facial Features & Intellectual Disability: Should prompt consideration of Kabuki syndrome (KMT2D/KDM6A), where diabetes arises from epigenetic dysregulation. Sensory Deficits (Vision/Hearing) [56]: Often precede or accompany diabetes in WFS1 (Wolfram) or GLIS3 mutations. By systematically screening for cardiac, renal, and sensory anomalies, clinicians can predict the genetic etiology and initiate targeted surveillance for comorbidities (e.g., renal ultrasound for HNF1B, cardiac echo for GATA6) [57].

#### 3.1.1. The Multi-System Developmental Syndromes: GLIS3, GATA6, and HNF1B

Mutations in transcription factors governing organogenesis frequently produce distinct “malformation syndromes”. GLIS3 mutations exemplify this category, causing a syndrome defined by Neonatal Diabetes, Congenital Hypothyroidism, and Congenital Glaucoma (NDH). Recent case reports have expanded this phenotype to include hepatic complications (cholestasis, hepatomegaly) and distinct ocular features like high hyperopia [58,59]. The hypothyroidism in these patients is typically congenital and requires immediate replacement therapy to prevent neurodevelopmental deficits. Moreover, the co-occurrence of renal cysts in GLIS3 deficiency highlights the gene’s role in maintaining renal tubular architecture, necessitating regular renal ultrasound monitoring [60].

Similarly, GATA6 and HNF1B mutations present with diabetes associated with structural defects in the heart and kidneys, respectively. GATA6 haploinsufficiency is the leading cause of pancreatic agenesis accompanied by congenital heart defects, most commonly Tetralogy of Fallot or atrial septal defects [61]. The diabetes is insulin-dependent from birth due to the critical reduction in β-cell mass. In contrast, HNF1B mutations (MODY5) cause Renal Cysts and Diabetes (RCAD) syndrome. While diabetes may present later in adolescence, early onset cases do occur, and the renal phenotype—ranging from cystic dysplasia to single kidneys—often precedes the metabolic dysfunction [62]. A recent study of HNF1B patients highlighted that genital tract malformations and electrolyte disturbances (e.g., hypomagnesemia) are also frequent, reinforcing the need for a comprehensive systemic workup [63]. A visual guide to the extra-pancreatic manifestations associated with specific monogenic forms of diabetes. Recognizing these “syndromic fingerprints” aids in rapid genetic prioritization. Neurological: KCNJ11 mutations can cause DEND syndrome (developmental delay, epilepsy) due to channel expression in the brain. Sensory: WFS1 is linked to optic atrophy and deafness; GLIS3 to congenital glaucoma; PAX6 to aniridia. Endocrine: GLIS3 causes congenital hypothyroidism. Cardiac: GATA6 haploinsufficiency is strongly associated with congenital heart defects (e.g., Tetralogy of Fallot). Renal/Gastrointestinal: HNF1B causes renal cysts and dysplasia; NEUROG3 leads to malabsorptive diarrhea.

#### 3.1.2. Metabolic and Neurosensory Syndromes: TRMA and Beyond

A unique subset of syndromic diabetes is caused by defects in metabolite transport rather than transcriptional regulation. Thiamine-Responsive Megaloblastic Anemia (TRMA) syndrome, caused by mutations in the thiamine transporter gene SLC19A2, presents with a classic triad: megaloblastic anemia, sensorineural deafness, and non-autoimmune diabetes [24]. The pathogenesis involves intracellular thiamine deficiency, which triggers endoplasmic reticulum stress and apoptosis in high-energy-demand tissues like hematopoietic cells and pancreatic β-cells [55]. Crucially, this is a potentially treatable form of syndromic diabetes; high-dose thiamine supplementation can ameliorate the anemia and, in some cases, improve glycemic control, although it has limited efficacy in reversing hearing loss [26,27]. Ophthalmological manifestations such as retinitis pigmentosa and optic atrophy have also been recently reported, suggesting a broader neuro-retinal involvement than previously recognized [28].

Other rare syndromes further illustrate the genetic complexity of congenital diabetes. Kabuki syndrome, typically associated with KMT2D or KDM6A mutations, is characterized by distinct facial features, intellectual disability, and growth retardation. Recent literature has identified that these patients are also at increased risk for autoimmune and non-autoimmune diabetes, likely due to the epigenetic dysregulation of β-cell function [29]. Additionally, syndromes involving DNA repair or ciliary function, such as Alström syndrome or DNAJC3-related neuroendocrine disorders, can present with early onset diabetes accompanied by neurodegeneration and sensory deficits [30]. These examples underscore that in any infant with diabetes and “atypical” features—be it anemia, deafness, or dysmorphic facies—a syndromic etiology must be suspected to facilitate early and targeted intervention.

### 3.2. Biphasic Phenotypes: The Transition from Hyperinsulinism to Diabetes

A particularly challenging and clinically significant subset of congenital diabetes involves a “biphasic” clinical trajectory. In these cases, the genetic defect initially manifests in the neonatal period as congenital hyperinsulinism (CHI), characterized by severe hypoglycemia and macrosomia. However, as the patient ages, the phenotype “flips” from insulin excess to insulin deficiency, eventually leading to the development of diabetes mellitus in childhood or early adulthood. Recognizing this biphasic pattern is crucial, as a history of neonatal hypoglycemia in a child presenting with diabetes is a strong predictor of specific monogenic etiologies, distinguishing them from autoimmune type 1 diabetes.

#### 3.2.1. The HNF4A and HNF1A Paradigm

The transcription factors HNF4A (MODY1) and HNF1A (MODY3) are best known as causes of maturity-onset diabetes of the young. However, they play a dual role in β-cell physiology. In fetal life, HNF4A and HNF1A are essential for regulating the expression of the channel subunit KCNJ11. Mutations in these genes can disrupt this regulation, leading to a transient reduction in channel function in utero. This results in fetal hyperinsulinemia and macrosomia (birth weight > 4000 g), followed by neonatal hypoglycemia [41,42].

As the child grows, the mechanism shifts. The persistent dysfunction of the transcription factors leads to a progressive failure in the maintenance of mature β-cell identity and function, causing the gradual onset of insulin deficiency. Recent studies have expanded this spectrum to include HNF1A, which was historically thought to lack the neonatal hypoglycemic phase seen in HNF4A. A 2020 cohort study confirmed that pathogenic HNF1A variants can indeed cause severe diazoxide-responsive hyperinsulinemic hypoglycemia in neonates, which later evolves into classic MODY diabetes [42]. Furthermore, novel case reports have identified HNF1A mutations presenting with complex phenotypes, including congenital hyperinsulinism accompanied by hepatomegaly and renal tubular dysfunction, suggesting that HNF1A-related disease can also be syndromic in nature [63]. Therefore, inquiring about birth weight and neonatal hypoglycemia is a mandatory component of the anamnesis for any antibody-negative young diabetic patient.

#### 3.2.2. The Paradox of KATP Channel Mutations

While gain-of-function mutations in ABCC8 and KCNJ11 cause neonatal diabetes, loss-of-function (LoF) mutations typically cause congenital hyperinsulinism. However, a “paradoxical” biphasic phenotype has been increasingly recognized in patients carrying specific LoF variants in these channel genes. These individuals present with hyperinsulinemic hypoglycemia in infancy but subsequently develop diabetes, often misdiagnosed as type 1 or type 2 diabetes later in life [64].

The mechanism underlying this transition is hypothesized to be β-cell “burnout”. The constitutive closure of the channel leads to persistent membrane depolarization and continuous calcium influx. Over years, this chronic overstimulation may induce endoplasmic reticulum stress, mitochondrial dysfunction, and ultimately apoptosis of the β-cells [44]. A groundbreaking study provided further mechanistic insight, demonstrating that certain channel mutations result in reduced surface expression of the Kir6.2/SUR1 complex [65]. While this initially mimics a channel blockade (causing hypoglycemia), the long-term inability of the β-cell to rest or modulate secretion leads to functional exhaustion and diabetes [34]. Clinically, this distinction is vital because these patients, despite having “diabetes”, may still retain some responsiveness to sulfonylureas or may require specific management to preserve remaining β-cell mass, although the optimal treatment window remains a subject of active research [55].

## 4. Diagnostic Strategy and Precision Medicine: From Molecular Identification to Targeted Therapy

### 4.1. Defining the Diagnostic Window and Algorithm

The diagnosis of neonatal diabetes mellitus (NDM) has fundamentally shifted from a clinical exclusion of type 1 diabetes to a proactive molecular investigation [16]. Historically, the “monogenic window” was strictly defined as diabetes onset before 6 months of age, a period during which autoimmune type 1 diabetes is exceptionally rare [27]. Consequently, immediate genetic testing is mandatory for all infants presenting with hyperglycemia within this timeframe [36,48]. However, recent large-scale cohort studies have blurred this chronological boundary [39,50], demonstrating that pathogenic variants in genes such as INS, ABCC8, and KCNJ11 can manifest between 6 and 12 months of age, or even later [31,32]. Therefore, the diagnostic algorithm must be expanded: genetic testing should be strongly considered for any infant diagnosed up to 12 months, particularly those who are negative for pancreatic autoantibodies (anti-GAD [33], anti-IA2, anti-ZnT8) and retain detectable C-peptide levels. Furthermore, the presence of specific syndromic features serves as a critical diagnostic filter independent of age; for instance, the identification of renal cysts, sensorineural deafness, or optic atrophy should trigger immediate genetic evaluation for syndromes like HNF1B-MODY or Wolfram syndrome, regardless of whether the diabetes onset fits the classical neonatal timeframe [34].

### 4.2. Methodological Evolution: Next-Generation Sequencing and Variant Interpretation

**Figure 3:**
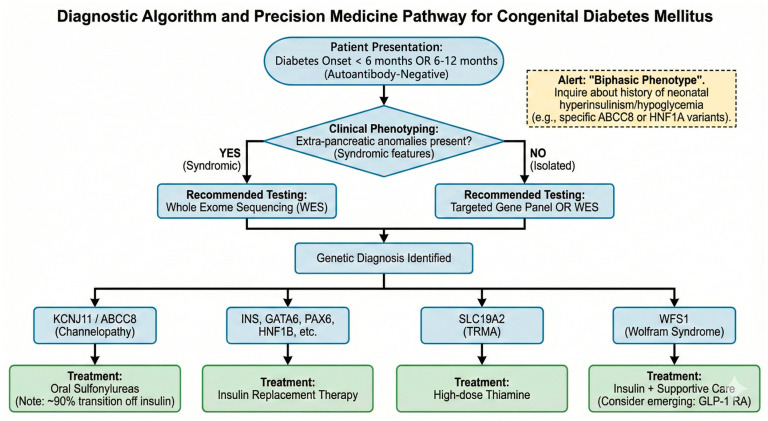
Integrated Diagnostic Algorithm and Precision Medicine Pathway.

The transition from candidate gene Sanger sequencing to Next-Generation Sequencing (NGS) has revolutionized diagnostic efficiency. While targeted gene panels remain a cost-effective and rapid first-line approach—crucial for quickly identifying KCNJ11 or ABCC8 mutations to facilitate early treatment transition—Whole Exome Sequencing (WES) is increasingly advocated for syndromic presentations or panel-negative cases [45]. WES allows for the detection of rare or novel variants in transcription factors (e.g., GLIS3, GATA6, NEUROG3) that might be omitted from standard panels [46]. However, this technological leap introduces the challenge of interpreting Variants of Uncertain Significance (VUS). A rigorous evaluation framework is essential, integrating bioinformatic prediction tools with detailed clinical phenotyping. For example, the pathogenicity of a VUS in HNF1B is strongly supported if renal imaging confirms cystic dysplasia. In cases where genotype–phenotype correlations are ambiguous, functional validation assays—such as patch-clamp electrophysiology for KATP channels or cellular assays for receptor misfolding—are becoming an indispensable component of the diagnostic workflow to prevent misdiagnosis and ensure appropriate therapeutic interventions [47], genetic testing is mandatory for all infants presenting with hyperglycemia within this timeframe (Figure 3). A stepwise approach to the management of neonatal diabetes. Step 1 (Screening): Identify infants with diabetes onset <6 months, or 6–12 months if antibody-negative. Step 2 (Phenotyping): Assess for syndromic features (refer to Figure 2) to guide testing choice (Targeted Panel vs. Whole Exome Sequencing). Step 3 (Diagnosis & Treatment): Upon confirming a genetic etiology, initiate mechanism-based therapy.

### 4.3. Pharmacogenomics: The Paradigm of Sulfonylurea Therapy

The most profound application of precision medicine in NDM is the treatment of KCNJ11 and ABCC8 channelopathies [48]. These mutations typically result in a “gain-of-function” defect where the potassium channel remains constitutively open, preventing membrane depolarization and insulin release despite preserved insulin synthesis [49]. High-dose oral sulfonylureas (e.g., glibenclamide) directly bind to the SUR1 subunit, closing the channel via an ATP-independent mechanism and restoring endogenous insulin secretion [50]. Clinical evidence overwhelmingly supports this transition; approximately 90% of patients with KCNJ11 mutations and 85% of those with ABCC8 mutations can successfully discontinue insulin injections. The benefits of this “molecular switch” extend far beyond glycemic control. Unlike exogenous insulin, sulfonylureas have been shown to stabilize glucose fluctuations and significantly lower HbA1c levels without increasing the risk of severe hypoglycemia. More importantly, for patients with DEND syndrome (Developmental delay, Epilepsy, and Neonatal Diabetes), sulfonylureas can cross the blood–brain barrier [51,52]. Recent studies confirm that early high-dose treatment can ameliorate neurological deficits [53], improving motor function and attention span—therapeutic outcomes that are unattainable with insulin therapy alone. Even in adult patients who have been misdiagnosed with type 1 diabetes for decades, genetic identification allows for a successful transfer to oral medication, demonstrating that β-cell function can be “rescued” after years of dormancy [54].

### 4.4. Beyond Channels: Emerging Therapies for Syndromic Forms

Precision medicine is also expanding to address complex syndromic forms of diabetes previously considered untreatable. In Thiamine-Responsive Megaloblastic Anemia (TRMA) syndrome, caused by SLC19A2 mutations, the primary defect is intracellular thiamine deficiency due to transporter failure. The administration of pharmacological doses of oral thiamine utilizes low-affinity transporters to restore intracellular levels, which can correct the megaloblastic anemia and significantly improve diabetes control, potentially eliminating the need for insulin in the early stages of the disease [16]. Similarly, the management of Wolfram syndrome (WFS1) is evolving from purely supportive care to mechanism-based disease modification. Recognizing that WFS1 deficiency drives β-cell apoptosis through chronic endoplasmic reticulum (ER) stress, recent preclinical and clinical investigations have highlighted the potential of GLP-1 receptor agonists (e.g., liraglutide) [17]. These agents have been shown to mitigate ER stress responses and protect mitochondrial function in WFS1-deficient cells [18]. Case reports suggest that GLP-1 receptor agonists may not only improve glycemic control but could also stabilize the neurodegenerative progression characteristic of this syndrome, marking a significant shift towards targeted therapies that address the root cellular pathology rather than just the symptoms19.

### 4.5. Genetic Counseling and Long-Term Surveillance

A definitive molecular diagnosis provides the necessary data for accurate genetic counseling and long-term prognosis, which varies dramatically by gene. For instance, KCNJ11 and INS mutations frequently arise de novo, implying a low recurrence risk for future siblings, whereas WFS1 and SLC19A2 follow an autosomal recessive pattern with a 25% recurrence risk, and HNF1B is typically autosomal dominant with a 50% risk. Understanding these inheritance patterns is vital for family planning and preimplantation genetic diagnosis. Furthermore, the specific genetic etiology dictates the surveillance strategy for extra-pancreatic comorbidities. A diagnosis of HNF1B mutation necessitates lifelong monitoring of renal function and magnesium levels due to the high risk of cystic dysplasia and electrolyte imbalances. Similarly, patients with GATA6 mutations require regular cardiac evaluations, while those with GLIS3 defects must be monitored for glaucoma and thyroid dysfunction. This genotype-first approach ensures that potentially life-threatening complications are identified and managed preemptively, optimizing the long-term quality of life for affected children [66].

## 5. Challenges and Future Perspectives

Despite the transformative advances in understanding the genetic basis of neonatal diabetes mellitus (NDM), significant hurdles remain in translating these discoveries into equitable clinical care. Furthermore, as the low-hanging fruit of channelopathies has been harvested, the field now faces the more arduous task of developing therapies for transcription factor defects and exploring the non-coding genome.

### 5.1. The Interpretation Bottleneck: Variants of Uncertain Significance (VUS)

The widespread adoption of Whole Exome Sequencing (WES) has created a data deluge, often identifying novel variants that lack definitive evidence of pathogenicity. These Variants of Uncertain Significance (VUS) pose a major dilemma: incorrect labeling can lead to inappropriate treatment cessation or unnecessary anxiety, while dismissing a pathogenic variant delays precision therapy. This challenge is particularly acute in genes like HNF1B or ABCC8, where phenotype variability is high. To address this, there is an urgent need to integrate high-throughput functional validation assays into the clinical workflow. A 2024 study demonstrated the utility of in vitro functional analysis in reclassifying HNF1B variants, proving that molecular assays can effectively resolve diagnostic ambiguity and guide clinical management [37]. Moving forward, the development of “variant maps” using CRISPR-based saturation mutagenesis could allow clinicians to look up the functional consequence of any given mutation instantly.

### 5.2. Global Disparities and Genomic Representation

Current genetic knowledge is heavily skewed toward populations of European descent. This bias limits the diagnostic yield for patients from other ethnic backgrounds, who may harbor population-specific variants not captured in standard reference databases. Recent initiatives in Asia have begun to bridge this gap. For instance, the establishment of an Asian monogenic diabetes registry has clarified the prevalence and renal trajectories of local patients, developing specific diagnostic algorithms that differ from Western guidelines [18]. Similarly, studies in Western Siberia have mapped unique mutation spectra for MODY-associated genes, emphasizing that genetic testing panels must be optimized for regional genetic diversity to ensure equitable access to precision medicine [29].

### 5.3. Beyond Small Molecules: Gene Therapy and Cell Replacement

While sulfonylureas have revolutionized the treatment of channelopathies, patients with transcription factor defects (e.g., PAX6, GATA6, GLIS3) or severe β-cell destruction currently lack targeted therapies and remain dependent on insulin. For these individuals, the future lies in gene therapy and regenerative medicine.

Recent breakthroughs in gene delivery offer hope. In 2023, researchers successfully used adeno-associated virus (AAV) vectors to deliver the PAX6 gene in diabetic models, demonstrating that replenishing this critical transcription factor can preserve β-cell mass and improve islet transplantation efficacy [30]. Similarly, gene therapy approaches are being explored for metabolic syndromes associated with lipodystrophy (e.g., BSCL2 mutations), where restoring gene expression has been shown to recover adipose tissue function and metabolic health in preclinical models [51]. For patients with pancreatic agenesis (e.g., GATA6 deficiency), stem cell-derived β-cell replacement combined with gene editing to correct the underlying mutation represents the ultimate curative horizon.

### 5.4. The Dark Matter of the Genome: Non-Coding RNAs and Epigenetics

The majority of NDM research has focused on the protein-coding exome. However, the non-coding genome plays a pivotal role in β-cell development and function. Emerging evidence suggests that long non-coding RNAs (lncRNAs) and microRNAs (miRNAs) act as fine-tuners of the insulin secretion machinery. A 2025 study identified that the lncRNA PAX6-AS1 (antisense to PAX6) is upregulated under hyperglycemic conditions and negatively regulates β-cell function, suggesting that targeting these RNA molecules could offer a novel therapeutic strategy [42]. Furthermore, miRNAs such as miR-365-3p have been found to inhibit the differentiation of mesenchymal stem cells into islet-like clusters by targeting PAX6, highlighting a complex regulatory network that extends beyond the DNA sequence [33]. Finally, epigenetic mechanisms, including multilocus imprinting disturbances, are increasingly recognized in syndromic forms of diabetes, adding another layer of complexity to the genetic diagnosis [46]. Future research must decipher this “epigenetic code” to fully understand the pathogenesis of idiopathic cases that remain unsolved by WES.

## 6. Conclusions

Neonatal diabetes mellitus has emerged from the shadow of type 1 diabetes to stand as a distinct and instructive model of monogenic metabolic disease. As this review has delineated, the condition is not a singular entity but a heterogeneous spectrum of disorders defined by specific molecular failures. The transition from a clinical diagnosis based solely on age of onset to a molecular diagnosis based on genetic etiology represents a fundamental paradigm shift in pediatric endocrinology.

Our understanding of the pathogenic mechanisms has evolved significantly. We now recognize three cardinal pathways driving β-cell failure: the electrophysiological uncoupling seen in channelopathies, the developmental arrest caused by transcription factor defects, and the irreversible cellular exhaustion driven by endoplasmic reticulum stress. The recognition of complex clinical trajectories, such as the biphasic transition from hyperinsulinism to diabetes, further underscores the dynamic nature of these genetic defects and the necessity for longitudinal vigilance.

Most importantly, the study of neonatal diabetes mellitus stands as a beacon for the promise of precision medicine. The ability to transition patients with KCNJ11 and ABCC8 mutations from insulin injections to oral sulfonylureas is more than a therapeutic success; it is a proof-of-concept that understanding the molecular “why” can fundamentally change the clinical “how”. Similarly, the potential repurposing of GLP-1 receptor agonists for Wolfram syndrome and thiamine supplementation for TRMA highlights that treatable targets exist even within complex syndromic presentations.

Looking forward, the field faces the dual challenge of expanding access to genetic diagnostics and developing curative therapies for those who do not respond to current oral agents. As next-generation sequencing becomes routine, the integration of functional validation for novel variants will be essential to bridge the gap between genotype and phenotype. Ultimately, the goal extends beyond glycemic control; it is to leverage genetic insights to predict comorbidities, offer accurate reproductive counseling, and pave the way for gene-editing therapies that may one day offer a permanent cure for these lifelong conditions. Through early suspicion, precise diagnosis, and mechanism-based management, clinicians can profoundly alter the natural history of congenital diabetes and improve the quality of life for affected children and their families.

## Figures and Tables

**Table 1:** Genotype–Phenotype Correlations and Therapeutic Strategies for K_ATP_ Channelopathies.

Genetic Defect	Mutation Type	Mechanism of Action	Clinical Phenotype	Primary Therapy
*KCNJ11*/*ABCC8*	Gain-of-Function (GoF)	Decreased ATP sensitivity or increased channel opening probability; constitutive hyperpolarization prevents insulin release.	Neonatal Diabetes (TNDM or PNDM)	Sulfonylureas
			DEND Syndrome (with neurological involvement)	(High-dose, binds SUR1 to force channel closure)
*ABCC8*/*KCNJ11*	Loss-of-Function (LoF)	Channel fails to open or traffic to membrane; constitutive depolarization leads to unregulated insulin secretion.	Congenital Hyperinsulinism (CHI)	Diazoxide (channel opener) or somatostatin analogues
			Persistent Hypoglycemia	
*ABCC8*/*KCNJ11*	Paradoxical LoF/Biphasic	Initial LoF causes β-cell exhaustion or apoptosis; or specific variants reduce membrane expression leading to eventual failure.	Biphasic: CHI in infancy →Diabetes in later life	Variable:
			MODY-like diabetes (e.g., MODY12)	Insulin or sulfonylureas (depending on residual β-cell mass)

**Table 2:** Differential Diagnosis of Isolated and Syndromic Neonatal Diabetes Mellitus.

Genetic Etiology	Primary Classification	Syndrome/Condition	Key Extra-Pancreatic Clinical Features
*KCNJ11*/*ABCC8*	Isolated (Functional)	PNDM/TNDM/DEND	Typically none (Isolated).
			DEND: Developmental delay, Epilepsy, Muscle weakness (Functional neurological, not structural) [55].
*INS*	Isolated	PNDM	None. Pure β-cell destruction or proinsulin misfolding.
*GLIS3*	Syndromic	NDH Syndrome	Congenital Hypothyroidism.
			Congenital Glaucoma & High Hyperopia [58].
			Renal cysts, Hepatic cholestasis [59,60].
*GATA6*	Syndromic	Pancreatic Agenesis	Congenital Heart Defects (Tetralogy of Fallot, ASD/VSD).
			Pancreatic exocrine insufficiency [61].
*HNF1B*	Syndromic	RCAD (MODY5)	Renal Cysts (Cystic dysplasia), Single kidney.
			Genital tract malformations (e.g., Uterine anomalies).
			Hypomagnesemia [62,63].
*SLC19A2*	Syndromic	TRMA Syndrome	Megaloblastic Anemia (Thiamine-responsive).
			Sensorineural Deafness.
			Retinitis pigmentosa/Optic atrophy [34,38].
*WFS1*	Syndromic	Wolfram Syndrome	Optic Atrophy.
			Sensorineural Deafness.
			Diabetes Insipidus, Neurodegeneration [43].
*PAX6*	Syndromic	Aniridia-Diabetes	Aniridia (Absence of iris).
			Cataracts, Nystagmus.
			Obesity [32,34].
*NEUROG3*	Syndromic	Enteric Endocrinopathy	Congenital Malabsorptive Diarrhea (Lack of enteroendocrine cells) [38].
*KMT2D*	Syndromic	Kabuki Syndrome	Distinctive facial features, Intellectual disability.
			Growth retardation, Immune abnormalities [64].

## Data Availability

No new data were created or analyzed in this study. Data sharing is not applicable to this article.
